# Next-generation sequencing of homologous recombination genes could predict efficacy of platinum-based chemotherapy in non-small cell lung cancer

**DOI:** 10.3389/fonc.2022.1035808

**Published:** 2022-12-14

**Authors:** Linlin Zhang, Shasha Guan, Fanlu Meng, Lin Teng, Diansheng Zhong

**Affiliations:** ^1^ Department of Medical Oncology, Tianjin Medical University General Hospital, Tianjin, China; ^2^ Hangzhou Jichenjunchuang Medical Laboratory Co. Ltd., Hangzhou, China

**Keywords:** NGS, homologous recombination, platinum sensitivity, chemotherapy, NSCLC

## Abstract

**Background:**

With the widespread use of next-generation sequencing (NGS) in clinical practice, an increasing number of biomarkers that predict a response to anti-tumor therapy in non-small cell lung cancer (NSCLC) has been identified. However, validated biomarkers that can be used to detect a response to platinum-based chemotherapy remain unavailable. Several studies have suggested that homologous recombination deficiency (HRD) may occur in response to platinum-based chemotherapy in ovarian cancer and breast cancer. However, currently there is a lack of proven and reliable HRD markers that can be used to screen for patients who may benefit from platinum-based chemotherapy, especially in NSCLC.

**Methods:**

NGS was used to screen for gene mutations, including homologous recombination (HR) genes and common driver gene mutations in NSCLC. Cox regression analysis was performed to identify potential clinicopathological or gene mutation factors associated with survival in patients receiving platinum-based chemotherapy, while Kaplan–Meier analysis with the log-rank test was performed to assess the effect of HR gene mutations on progression-free survival (PFS).

**Results:**

In a retrospective cohort of 129 patients with advanced NSCLC, 54 who received platinum-based chemotherapy with or without anti-angiogenic therapy were included in the analysis. Univariate and multivariate Cox proportional hazard regression analyses showed that HR gene mutations were associated with platinum-based chemotherapy sensitivity. Efficacy results indicated that the objective response rates (ORR) for patients with *BRCA1/2* mutations and *BRCA1/2* wild type were 75% and 30.4% (*p*=0.041), while the median PFS was 7.5 and 5.5 months (hazard ratio [HR], 0.52; 95% CI, 0.27–1.00; *p=*0.084), respectively. The ORRs of patients with HR gene mutations and HR gene wild type were 60% and 23.6% (*p*=0.01), and the median PFS was 7.5 and 5.2 months (HR, 0.56; 95% CI, 0.32–0.97; *p=*0.033), respectively.

**Conclusions:**

HR gene mutations show potential as promising biomarkers that may predict sensitivity to platinum-based chemotherapy in advanced and metastatic NSCLC.

## Introduction

Globally, lung cancer is the most prevalent and the second most prevalent cause of cancer-related deaths in men and women, respectively (1, 2). It is also the most frequent cancer and the leading cause of cancer-related deaths in China (3). Non-small cell lung cancer (NSCLC) accounts for approximately 80–90% of lung cancers (4), mainly including a variety of histological types, such as squamous cell carcinomas, adenocarcinoma, and large cell carcinoma.

Targeted therapies and immunotherapies have been widely used to treat NSCLC in recent years (5–7). Approximately 60%–70% of adenocarcinoma carry targetable oncogenic driver molecular alterations. While targeted therapies are possible (8), drug resistance to targeted therapies is inevitable. Only a minority (less than 20%) of unselected NSCLC patients respond to immunotherapies, and some of these patients suffer from severe immunotoxicity (9). Platinum-based chemotherapies, which focus on DNA damage in tumor cells, are still a part of the backbone treatment regimen for NSCLC (10). Therefore, screening for chemotherapy-benefit populations would certainly enhance clinical practice.

BRCA1/2 mutations are known to trigger a DNA damage response (DDR), and DDR pathway deficiencies may drive genomic instability, thereby affecting tumorigenesis and progression (11). Previous studies have suggested that high expression levels of some DDR genes are associated with poor response to platinum-based chemotherapy in NSCLC patients (12–14). However, to date, no proven reliable DDR markers have been made available for the purpose of detecting patients who would benefit from platinum-based chemotherapy (15, 16).

Homologous recombination (HR), a sub-process of DDR, is the most important mechanism associated with DNA double-strand break repair. Deficiencies in the HR pathway may theoretically lead to higher genomic instability and sensitivity to platinum (17). The widespread use of next-generation sequencing (NGS) in clinical genetic detection in recent years, has resulted in an increasing number of studies which indicate that homologous recombination deficiency (HRD) may be used for predicting the response of ovarian cancer, breast cancer, and certain other tumors to platinum-based chemotherapies (18–21).

Several studies have shown that, in NSCLC, HR gene mutations, expression levels, and HRD scores are associated with the response to platinum-based chemotherapy (22–25). However, largely due to sample size limitations, these studies do not accurately reflect the actual relationship between HRD genes and the platinum sensitivity of NSCLC. Therefore, in this study, we focused on genetic variations in the HR pathway and sought to describe the clinical characteristics of patients with deleterious HR gene mutations, while investigating whether gene mutations in the HR pathway correlate with sensitivity to platinum-based chemotherapy in advanced NSCLC patients.

## Materials and methods

### Subject selection

The study cohort consisted of NSCLC patients treated at the medical oncology department of Tianjin Medical University General Hospital (Tianjin, China), between Dec 2018 and July 2020. The participating patients underwent genetic screening *via* NGS before receiving treatment. Samples collected from patients included blood and tumor tissues. Of the 54 patients included in the efficacy analysis, tumor tissue samples were collected from 31, while blood samples were collected from 23 whose tumor samples could not be accessed. Patients with small-cell lung cancer or other metastatic pulmonary malignancies were excluded from the study. Patients who received standard treatment with platinum chemotherapy, with or without anti-angiogenic agents, were enrolled in the efficacy analysis. However, patients who received platinum-based chemotherapy combined with immunotherapy were excluded. The final follow-up period was January 2022.

### Ethics approval and consent to participate

The experimental protocol was established according to the ethical guidelines of the Helsinki Declaration and approved by the Human Ethics Committee of Tianjin Medical University General Hospital. The ethical approval number is IRB2019-075-01. Written informed consent was obtained from individual participants or the guardians of participants.

### Sample handling, DNA extraction and next-generation sequencing

Five paraffin sections (5-µm thick) used as tissue samples were transported at room temperature. Intravenously obtained blood samples (10-mL each) were collected in Streck tubes, transported at room temperature, and centrifuged to separate the plasma and leukocytes.

Genomic DNA was isolated from tumor tissues and leukocytes using an AllPrep DNA/RNA mini Kit (Qiagen Sciences, Germantown, MD, USA), where leukocyte DNA was used as a germline control. Cell-free plasma DNA was extracted using a MagMAX™ Cell-Free DNA Isolation Kit (Thermo Fisher Scientific, Waltham, MA, USA). Isolated DNA was then qualified for fragment size, quality, and concentration, among other criteria, using a 2200 Bioanalyzer (Agilent Technologies, Palo Alto, CA, USA).

Genomic and circulating tumor DNA (ctDNA) libraries were constructed using a KAPA Hyper Prep kit (KAPA Biosystems, Wilmington, MA, USA) according to the manufacturer’s instructions, and captured with a gene panel (in-house 831-gene panel of Genetron Health Co. Ltd.) that covered a majority of tumor-related genes and HR genes. A list of HR genes is shown (**Supplementary Table 1**). The libraries were sequenced using an Illumina NovaSeq 6000 system.

### Bio-informatics analysis

Primary processing of raw sequences included quality control *via* FASTQC (v0.11.9), and demultiplexing and masking of dual-index adapter sequences using Trimmomatic (v0.36). Sequence reads were then aligned to the human reference genome (version GRCh37/hg19) with BWA (v0.7.10). Quality control criteria used for sequencing and mutation calling were as follows: for tumor tissue samples - average sequencing depth after deduplication ≥500, and coverage of regions with sequencing depths reaching 140× ≥90%; for normal leukocyte samples - average sequencing depth after deduplication ≥250× and coverage of regions with sequencing depth reaching 10× ≥90%. When calling somatic mutations, the number of unpaired mutation reads at single nucleotide variation/insertion-deletion (SNV/InDel) positions ≥7, variant allele frequency (VAF) of hotspot mutation ≥1%, and the VAF of non-hotspot mutation ≥5% were considered. For ctDNA samples, the average sequencing depth was ≥10,000× before deduplication and ≥1,000× after deduplication, while for normal leukocyte samples, the average depth after deduplication was ≥100× and coverage of regions with sequencing depth reaching 10× ≥90%. When calling somatic mutations, the number of paired mutation reads at SNV/InDel positions ≥4, and the VAF of SNV/InDel ≥0.1% were considered.

For tumor tissue samples, somatic SNVs and InDels were called by comparing with matched leukocyte sequencing data using Mutect (version 3.1) and Strelka (version 2.9.2), respectively (26). For plasma samples, somatic SNVs and InDels of ctDNA were called using samtools (version 1.3.1) and pindel (version 0.2.5b8) across the targeted regions of interest. The effects of the variants were annotated using Oncotator (v1.5.1.0) and Variant Effect Predictor (VEP, version92). Variants were then filtered using the GTH false positive database (an in-house database of Genetron Co. Ltd.), the 1000 Genome Project database (https://www.internationalgenome.org/), the NHLBI Exome Sequencing Project (ESP, https://evs.gs.washington.edu/EVS/) and the gnomAD database (http://gnomad-sg.org/). Integrative Genomics Viewer (IGV) was applied to filter alignment and sequencing artifacts. Germline variants in leukocyte samples were identified by comparing with the human reference genome (version GRCh37/hg19) using HaplotypeCaller following GATK best practice (v3.5-0-g36282e4). The variants were then filtered as follows: filter out intron and splice mutations and retrieve pathogenic or pathogen-like intron and splice mutations according to the PLP_intron.list database (an in-house database of Genetron Co. Ltd.); filter out mutations with frequencies less than 20% and total depths less than 5.

All nonsense, deletion, splice site, and frameshift mutations of HR genes were defined as deleterious mutations. Deleterious missense mutations were defined as those reported as being pathogenic by the Catalogue Of Somatic Mutations In Cancer (COSMIC) or ClinVar, or those showing a Polyphen-2 score of ≥0.95. Patients carrying one or more deleterious HR gene mutations were considered as HR-mutant, while those without deleterious HR gene mutations were considered as HR-wild type.

### Clinical data collection

Clinical data, including age, sex, smoking status, histopathology, tumor stage, and treatments, were collected. To identify the relationship between platinum response and HR gene mutations, follow-up information of patients who received platinum-based chemotherapy with or without anti-angiogenic therapy was collected. Tumor responses to platinum-based treatments were evaluated using the RECIST 1.1 guideline. Progression-free survival (PFS) was defined as the time period between treatment initiation and tumor progression.

### Statistical analysis

A Cox proportional hazards regression model was used to ascertain the association between individual clinicopathological factors and survival. A comparison between the PFS times of *BRCA1/2*, HR wild-type and mutated groups was conducted using log-rank tests and visualized using Kaplan Meier plots. All statistical analyses were performed using SPSS Statistics version 26.0 (SPSS Inc., Chicago, IL, USA). A two-sided P value of 0.05 or less was considered statistically significant.

## Results

### Baseline information regarding NSCLC patients and the treatment regimen

We recruited 129 NSCLC patients from December 2018 to July 2020, 54 of whom had received platinum-based chemotherapy. Clinicopathological characteristics of the 54 patients who had received platinum-based chemotherapy were collected. These 54 patients included 19 women (35.2%) and 35 men (64.8%) with a median age of 64 years, ranging from 31–77 years (**Table 1**). Two-thirds (68.5%) of the patients (n = 37) had a history of smoking, and most had stage IV disease (n = 52; 96.3%). Almost all patients had adenocarcinoma (n = 33; 61.1%) or squamous carcinoma (n = 20; 37.0%), except for one who had pulmonary sarcomatoid carcinoma (n = 1; 1.9%). Two-thirds of the patients had received platinum-based chemotherapy alone (n = 37, 68.5%), while the rest had received chemotherapy combined with anti-angiogenic agents (n =17, 31.5%). In addition to the treatments described above, a small number of patients had received tyrosine kinase inhibitor (TKI) therapy before chemotherapy (n = 7, 13.0%).

**Table 1:** Clinicopathological characteristics of the 54 NSCLC included in efficacy analysis.

Clinicopathological characteristic		Number of case (%)
Age, years		
Median (min-max)		64 (31–77)
≥65, N (%)		24 (44.4%)
<65, N (%)		30 (55.6%)
Sex, N (%)		
Male		35 (64.8%)
Female		19 (35.2%)
Smoking status, N (%)		
Never smoker		17 (31.5%)
Ex-smoker		7 (13.0%)
Smoker		30 (55.5%)
Histopathology, N (%)		
Adenocarcinoma		33 (61.1%)
Squamous carcinoma		20 (37.0%)
Sarcomatoid carcinoma		1 (1.9%)
Stage, N (%)		
IIIB		2 (3.7%)
IV		52 (96.3%)
Treatment, N (%)		
Chemotherapy		37 (68.5%)
Chemotherapy combined with anti-angiogenic therapy		17 (31.5%)
TKI-targeted therapy before chemotherapy, N (%)		
Yes		7 (13.0%)
No		47 (87.0%)

### Frequency of mutation defects

**Figure 1:**
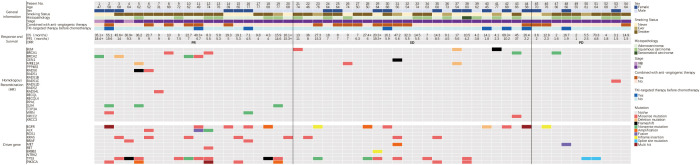
Baseline information of HR gene and driver gene mutations and the response to platinum-based chemotherapy in 54 NSCLC patients. The top panel showed clinical information and treatment. The middle panel showed response and survival, and the symbol ‘+’ after survival time indicated that the follow-up endpoint was not reached. The two panels below showed the HR and driver gene mutations. PFS-Progression free survival; ORR-Objective response rate; PR-Partial response; SD-Stable disease; PD-Progression disease.

Our retrospective evaluation of 54 NSCLC patients who had received platinum-based chemotherapy revealed 20 carrying HR gene mutations, eight carrying *BRCA1/2* mutations, and 22 carrying driver gene mutations (**Table 2**). Detailed mutation information is shown (**Figure 1**).

**Table 2:** HR gene and driver gene mutation information of 54 NSCLC patients.

Mutation type		Number of cases (%)
*BRCA1/2* mutation, N (%)		
Yes		8 (14.8%)
No		46 (85.2%)
HR gene mutation, N (%)		
Yes		20 (37.0%)
No		34 (63.0%)
Driver gene mutation, N (%)		
Yes		22 (40.7%)
No		32 (59.3%)

Our retrospective cohort of 129 NSCLC patients revealed 17 (13.2%) carrying *BRCA1/2* mutations, five carrying germline HR gene mutations, and 33 carrying somatic HR gene mutations. To identify deleterious mutations, we reviewed missense mutations in the COSMIC and ClinVar databases and analyzed them using Polyphen-2 software. The results indicated that six patients carrying *BRCA1* deleterious mutations and six patients carrying *BRCA2* deleterious mutations, among whom one carried both *BRCA1* and *BRCA2* mutations. All HR gene mutations in the 38 patients were deleterious (**Supplementary Figure 1**). The clinical and molecular characteristics of patients carrying *BRCA1/2* or HR gene mutations are shown (**Supplementary Tables 2**–**4**).

### Cox proportional hazard regression analysis of potential factors associated with progression-free survival and overall survival

**Figure 2:**
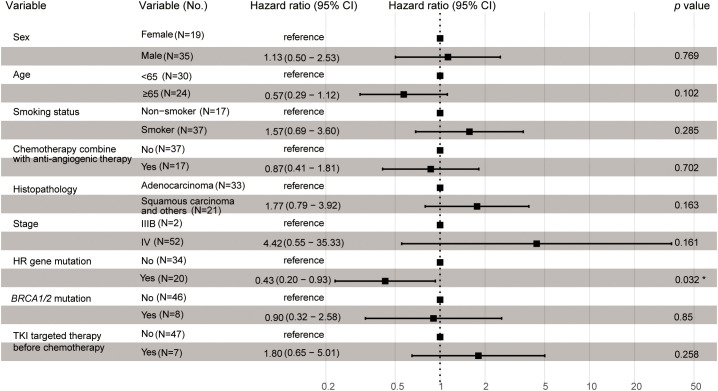
Multivariate Cox proportional hazard regression analysis of potential factors associated with progression-free survival. The asterisk indicated a statistically significant difference, that was, the p-value is less than 0.05.

Univariate and multivariate Cox regression analyses were performed to identify the association between potential factors and PFS or overall survival (OS). Univariate Cox regression analysis of patients who had received platinum-based chemotherapy showed that the HR gene mutation status was associated with their PFS (hazard ratio [HR], 0.54; 95% CI, 0.3–0.97; *p*=0.041), but not with their OS (HR, 0.75; 95% CI, 0.4–1.4; *p*=0.37) (**Tables 3**, **4**). When each HR gene was considered separately, the results indicated that any individual HR gene was not significantly associated with either PFS or OS, except for *RAD51C* (**Supplementary Table 5**). Since the particularly low mutation frequency of *RAD51C* (1/54) in our cohort may have led to analysis bias, it was not included in the multivariate analysis. Similarly, a statistically significant association with PFS or OS was not observed when each driver gene was considered as a separate factor (**Tables 3**, **4**). Multivariate analysis showed that HR gene mutation was significantly associated with PFS (HR, 0.43; 95% CI, 0.20-0.93; *p*=0.032), but not with OS (HR, 0.63; 95% CI, 0.28–1.41; *p*=0.26) (**Tables 3**, **4**; **Figure 2**). In addition, univariate Cox regression analysis showed that adenocarcinoma patients showed better OS following platinum-based chemotherapy (HR, 3.4; 95% CI, 1.8–6.3; *p*=0.00013), while multivariate analysis also yielded a similar result (HR, 4.34; 95% CI, 1.79–10.51; *p*=0.001) (**Table 4**).

**Table 3:** Univariate and multivariate Cox proportional hazard regression analysis of potential factors associated with progression-free survival.

Category		Univariate		Multivariate	
		HR (95% CI)	*p* value	HR (95% CI)	*p* value
Age ( ≥65 vs. <65)		0.87 (0.49-1.5)	0.62	0.57 (0.29-1.12 )	0.102
Sex (Male vs. Female)		1.1 (0.62-2)	0.71	1.13 (0.50-2.53)	0.769
Smoking status (Smoker vs. Non-smoker)		1.4 (0.75-2.6)	0.3	1.57 (0.69-3.60)	0.285
Histopathology (Squamous carcinoma and others vs. Adenocarcinoma)		1.5 (0.84-2.6)	0.18	1.77 (0.79-3.92)	0.163
Stage( IV vs. IIIB )		4.3 (0.59-31)	0.15	4.42 (0.55-35.33)	0.161
Combined with anti-angiogenic therapy (Yes vs. No)		0.85 (0.47-1.6)	0.6	0.87 (0.41-1.81)	0.702
TKI-targeted therapy before chemotherapy (Yes vs No)		1 (0.46-2.3)	0.95	1.80 (0.65-5.01)	0.258
HR gene mutation (Yes)		0.54 (0.3-0.97)	0.041 *	0.43 (0.20-0.93)	0.032 *
*BRCA1/2* mutation (Yes)		0.48 (0.2-1.1)	0.094	0.90 (0.32-2.58)	0.85
Driver gene mutation (Yes)					
*EGFR*		1.3 (0.61-2.6)	0.54		
*ALK*		1.1 (0.35-3.6)	0.85		
*ROS1*		1.5 (0.2-11)	0.24		
*KRAS*		0.54 (0.21-1.4)	0.19		
*BRAF*		0.98 (0.24-4.1)	0.98		
*MET*		1.1 (0.15-8.3)	0.9		
*RET*		1.5 (0.2-11)	0.7		
*ERBB2*		0.16 (0.022-1.2)	0.077		
*NTRK2*		1.1 (0.15-8.3)	0.9		
*TP53*		0.97 (0.54-1.7)	0.92		
*PIK3CA*		0.71 (0.3-1.7)	0.45		

The asterisk indicated a statistically significant difference, that was, the p-value is less than 0.05.

**Table 4:** Univariate and multivariate Cox proportional hazard regression analysis of potential factors associated with overall survival.

Category		Univariate		Multivariate	
		HR (95% CI)	*p* value	HR (95% CI)	*p* value
Age ( ≥65 vs <65)		1.7 (0.94-3.1)	0.076	1.63 (0.81-3.28)	0.17
Sex (Male vs Female)		1.3 (0.69-2.4)	0.41	0.88 (0.36-2.11)	0.77
Smoking status (Smoker vs Non-smoker)		1.3 (0.69-2.5)	0.4	0.92 (0.4-2.1)	0.84
Histopathology (Squamous carcinoma and others vs Adenocarcinoma)		3.4 (1.8-6.3)	0.00013*	4.34 (1.79-10.51)	0.001*
Stage (IV vs IIIB)		7.5e+07 (0-Inf)	1	3.43e+07 (0-Inf)	1
Combined with anti- angiogenic therapy (Yes vs No)		0.87 (0.47-1.6)	0.67	1.91 (0.83-4.38)	0.12
TKI-targeted therapy before chemotherapy (Yes vs No)		0.48 (0.18-1.2)	0.13	0.55 (0.16-0.88)	0.34
HR gene mutation (Yes)		0.75 (0.4-1.4)	0.37	0.63 (0.28-1.41)	0.26
*BRCA1/2* mutation (Yes)		0.53 (0.21-1.4)	0.19	0.58 (0.18-1.88)	0.37
Driver gene mutation (Yes)					
*EGFR*		0.61 (0.27-1.4)	0.25		
*ALK*		1.2 (0.38-4)	0.73		
*ROS1*		7.1 (0.87-57)	0.068		
*KRAS*		0.46 (0.16-1.3)	0.14		
*BRAF*		0.64 (0.087-4.7)	0.66		
*MET*		3.8e-08 (0-Inf)	1		
*RET*		3.3 (0.43-25)	0.25		
*ERBB2*		0.27 (0.037-2)	0.2		
*NTRK2*		3.8e-08 (0-Inf)	1		
*TP53*		0.79 (0.42-1.5)	0.47		
*PIK3CA*		0.92 (0.39-2.2)	0.85		

The asterisk indicated a statistically significant difference, that was, the p-value is less than 0.05.

### Predictive value of HR gene mutations and *BRCA1/2* mutations pertaining to platinum sensitivity in NSCLC

**Figure 3:**
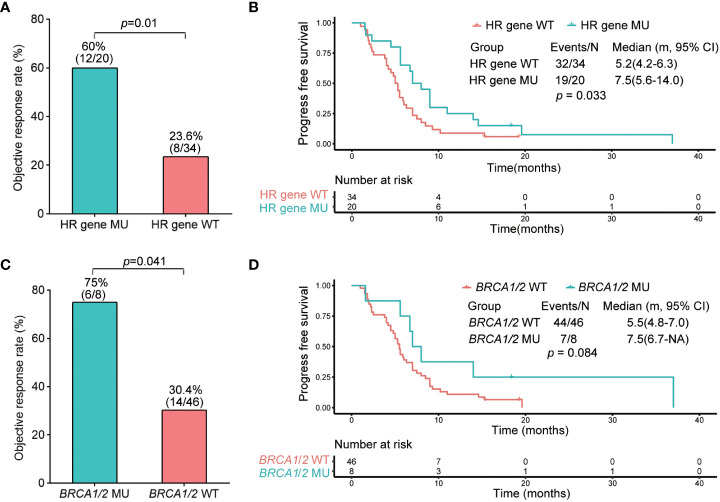
Objective response rate and progression-free survival analysis of HR and *BRCA1/2* gene wild type and mutated groups. **(A)** Objective response rate of HR gene wild type and mutated groups. **(B)** Kaplan–Meier analysis of progression-free survival of HR gene wild type and mutated groups. **(C)** Objective response rate of *BRCA1/2* gene wild type and mutated groups. **(D)** Kaplan–Meier analysis of progression-free survival of *BRCA1/2* gene wild type and mutated groups.

Fifty-four of the 129 patients who received standard platinum-based chemotherapy with or without anti-angiogenic agents were included in the analysis. We found that gene mutations/co-mutations in HR were correlated with the response shown by patients subjected to platinum-based chemotherapy. In addition, we divided patients displaying HR gene mutation status into two groups: HR gene wild-type (HR gene WT, n=34); and HR gene mutated (HR gene MU, n=20). The objective response rates (ORR) of HR gene WT and HR gene MU groups were 23.6% and 60% (*p*=0.01), respectively (**Figure 3A**). The median PFS of HR gene WT and HR gene MU were 5.2 months (4.2–6.3) and 7.5 months (5.6–14.0), respectively (HR, 0.56; 95% CI, 0.32–0.97; *p*=0.033); (**Figure 3B**).

Furthermore, because *BRCA1/2* is an important HR gene, we explored the correlation between *BRCA1/2* and platinum-based chemotherapy sensitivity. For this purpose, we divided the patients concerned into two groups: the *BRCA1/2* wild-type group (*BRCA1/2* WT, n=46); and the *BRCA1/2* mutated group (*BRCA1/2* MU, n=8). The ORR of patients in *BRCA1/2* WT and *BRCA1/2* MU were 30.4% and 75%, respectively (*p* = 0.041); (**Figure 3C**). The median PFS of *BRCA1/2* WT and *BRCA1/2* MU were 5.5 months (4.8–7.0) and 7.5 months (6.7-NA), respectively (HR, 0.52; 95% CI, 0.27–1.00; *p*=0.084), indicating that there was no significant difference between the two groups (**Figure 3D**).

### Relationship between HR gene mutations and clinical factors

**Figure 4:**
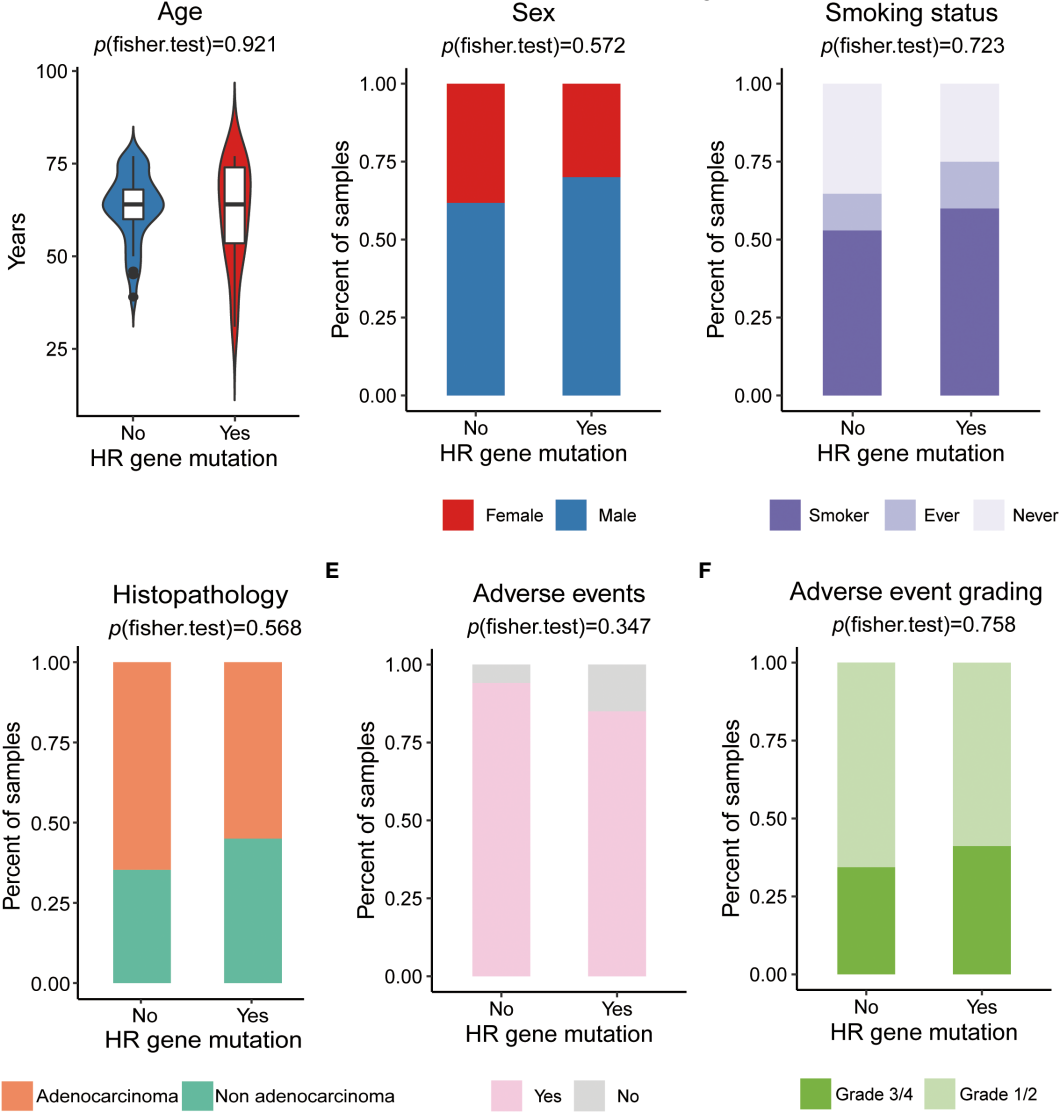
The relationship of HR gene mutations and clinical factors **(A)** Comparison of ages between HR mutation and wild-type groups. **(B)** Comparison of sex proportion differences in patients between HR gene mutation and wild-type groups. **(C)** Comparison of the differences in proportion of patients with a history of smoking between the HR gene mutation and wild-type groups. **(D)** Comparison of the differences in the proportion of patients related to histopathology type between the HR gene mutation and wild-type groups. **(E)** Comparison of the differences in the proportion of patient related to the occurrence of adverse events between the HR gene mutation and wild-type groups. **(F)** Comparison of the differences in the proportion of patients related to the incidence of grade 3/4 adverse events between the HR gene mutation and wild-type groups.

To investigate whether HR gene mutations are associated with clinical factors, we compared the differences between the proportions of patients in the HR gene mutation and wild-type groups, showing different clinical characteristics, including age, sex, smoking, and histopathology. The results indicated that there was no significant difference in age (*p*=0.927), sex (female vs. male, *p*= 0.572), smoking status (*p*= smoker vs. ex-smoker vs. never smoker, *p*=0.723), or histopathology (adenocarcinoma vs. non adenocarcinoma, *p*= 0.568) between HR gene mutation and wild-type groups, showing that there was no correlation between the HR gene mutations and clinical characteristics (**Figures 4A–D**).

Exploration of biomarkers that predict treatment efficacy requires taking treatment-related toxicities into consideration. The main treatment-related toxicities of 54 NSCLC patients who received platinum-based chemotherapy are summarized (**Supplementary Table 6**). Most patients (n=49, 90.7%) experienced treatment-related toxicities. The most common toxicities were myelosuppression (n=33, 61.1%), gastrointestinal reactions (n=21, 38.9%), and fatigue (n=26, 48.1%). Myelosuppression mainly manifested as neutropenia, leukopenia, and thrombocytopenia, while gastrointestinal reactions mainly manifested as nausea and vomiting. All platinum-based chemotherapy-related toxicities were common and manageable. To investigate whether HR genes were associated with treatment-related toxicities, we compared the occurrence of adverse events (AEs) and adverse event grading between HR gene mutation and wild-type groups, and found no significant differences between the occurrence of adverse events (yes vs. no, *p*=0.347), or between the incidence of grade 3/4 adverse reactions (grade 1/2 vs. grade 3/4, *p*=0.758); (**Figures 4E, F**; **Supplementary Table 7**). This indicated that HR gene mutations were not associated with treatment-related toxicities, suggesting that they could be used to predict chemotherapy sensitivity without increasing the incidence of adverse events.

## Discussion

Homologous recombination repair (HRR) and non-homologous end joining (NHEJ) are the major mechanisms underlying the repairing of DNA double-strand breaks (DSBs). HRR is a conservative process with high fidelity as it accurately repairs DNA damage without initiating any mutations. If HRR is functionally defective, resulting in homologous recombination deficiency (HRD), DSB repair is accomplished *via* NHEJ, which, unlike HRR, executes the repair by directly ligating the ends of the DSB, thus introducing deletions or mutations of DNA sequences at or around the site of DSB (11). The accumulation of genomic abnormalities leads to a higher level of genomic instability, which promotes tumorigenesis and progression and manifests as “genomic scarring,” which can be used as biomarkers of therapeutic response (27). HRD has been identified as a potential predictive biomarker in PARP inhibitor therapy against high-grade serous ovarian carcinoma (HGSOC), as well as in breast and prostate cancers (28–30).

The antitumor mechanism of platinum involves the establishment of covalent intra- and inter-strand cross-linking (ICL) between purine bases of DNA, which inhibits DNA replication and leads to cell death (31). Therefore, we focused on the role played by HR genes in platinum-based chemotherapy sensitivity of cancer. NGS has been commonly used for molecular typing of NSCLC, and the profiling of HR genes using NGS can be easily applied and achieved. Thus, we analyzed our NGS results to determine whether mutations or co-alterations of genes in the HR pathway may predict platinum sensitivity in NSCLC.

We aimed to identify whether PFS or OS may be associated with factors such as age, sex, smoking status, histopathology, tumor stage, combined with anti-angiogenic therapy status, TKI-targeted therapy before chemotherapy status, HR gene mutation, *BRCA1/2* mutation, and driver gene mutation status. Univariate and multivariate Cox regression analyses were performed. We found that the histopathological subtype of NSCLC was associated with OS (**Table 4**). This result was similar to those of previous studies. Several recent studies have indicated that OS among adenocarcinoma patients is significantly better than that among squamous cell carcinoma patients (32, 33). In addition, differences in the responses to certain chemotherapeutic regimens have been reported; for example, cisplatin combined with gemcitabine was more effective in squamous cell carcinoma, whereas cisplatin combined with pemetrexed was considered more effective for adenocarcinoma (34). Some somatic variants of certain genes, including *EGFR*, *ALK*, *ROS1*, *KRAS*, *BRAF*, *RET*, *MET*, *ERBB2*, *NTRK*, *TP53*, and *PI3KCA*, have been identified as common driver mutations in the NSCLC National Comprehensive Cancer Network (NCCN) guidelines (version 3.2022). Univariate and multivariate Cox regression analyses indicated that no single driver gene was associated with PFS or OS, and that, in a small number of patients, treatments targeting these driver genes were not associated with chemotherapy sensitivity. This is in line with clinical practice, because targeted drugs are prioritized when available.

*BRCA1/2* are important genes that affect HR ability, and *BRCA1/2* mutations have been used to predict platinum sensitivity in ovarian and breast cancers (35). Previous studies have also reported findings of NSCLC patients carrying *BRCA1/2* mutations, where 7 out of 8 patients carrying *BRCA1/2* mutations responded to platinum compounds (22). In our study, Cox regression analysis did not detect a significant association between *BRCA1/2* mutations and PFS or OS. However, we found that the ORR of patients carrying *BRCA1/2* mutations who were treated with platinum-based chemotherapy was 75%, which was significantly higher than that of patients carrying wild-type *BRCA1/2.* However, we did not find a significant difference between the PFS rates of *BRCA1/2* mutant and wild-type patients (**Figures 3C, D**). This result suggested that mutations in *BRCA1/2* alone might not be suitable for predicting platinum sensitivity, and that the role played by HR gene mutations should be considered.

The platinum response predictive value of HR gene mutations, which, in turn, is based on the platinum chemotherapy mechanism, has attracted increasing attention, particularly in regard to ovarian (36) and breast cancers (37, 38). Thus, we analyzed the association between HR gene mutations and sensitivity to platinum-based chemotherapy in NSCLC. Univariate Cox analysis showed that there was no significant correlation between individual genes of the HR pathway and PFS and OS, except for *RAD51C* (**Supplementary Table 5**). However, only one of the 54 patients harbored the *RAD51C* mutation. Genes with such a low mutation frequency cannot be used as representative predictive factors. Other HR genes showed no significant correlation with PFS and OS, which may be attributed to the small size of our cohort and the low mutation frequencies of each gene in the population. But when these HR genes were analyzed together, univariate and multivariate Cox regression analyses of PFS and OS suggested that they may play an important role in platinum response. The patients carrying HR gene mutations showed a higher response to platinum-based chemotherapy and a longer PFS (**Figures 3A, B**). Certain types of cancers, such as ovarian cancer (39), pancreatic cancer (40), and triple-negative breast cancer (37) that are treated with platinum-based chemotherapy as the first-line standard treatment usually show a higher frequency of HR deficiency. The results of this study, as well as those of previous studies, indicate that analysis of mutations in HR genes using NGS presents a promising and practical method that may be used to predict the response to platinum-based chemotherapy in NSCLC. On the other hand, these results also indicate that HR pathway gene mutations may play important roles in NSCLC tumorigenesis and malignant transformation, as they increase genomic instability. Many mechanistic studies have shown that driver mutations play an important role in NSCLC tumorigenesis and progression. Some have even been used as treatment targets in clinical settings (41, 42). Thus, our results indicate that the roles played by both classical driver mutations as well as HR gene mutations deserve equal attention when clinically treating NSCLC.

Application of a biomarker to predict treatment efficacy in clinical practice requires its effect on treatment-related toxicity to be taken into consideration. In our study, we observed that HR gene mutations did not lead to an increase in adverse events (**Figure 4E**). In addition, HR gene mutations did not significantly correlate with any of the specific clinical features (**Figures 4A–D**), suggesting that they may be used in clinical practice as promising biomarkers that predict chemotherapy sensitivity.

In our retrospective cohort of 129 NSCLC patients, the frequencies of *BRCA1/2* and HR mutations were 13.2% (17 of 129) and 29.5% (38 of 129), while the frequencies of deleterious *BRCA1/2* and HR mutations were 8.5% (11 of 129) and 29.5% (38 of 129), respectively, suggesting that approximately 30% of patients with advanced NSCLC may benefit from chemotherapy. To assess the proportion of advanced NSCLC patients who benefited from chemotherapy in the larger cohort, we counted the mutation frequencies of *BRCA1/2* and HR genes using two public databases. One was the Cancer Genome Atlas (TCGA) database, containing information for 515 lung adenocarcinomas and 485 lung squamous cell carcinoma mutations, while the other was OncoSG, containing information for 302 East Asian lung adenocarcinoma mutations (43). In the TCGA database, we detected 109 (10.9%) *BRCA1/2* mutation carriers, of which 73 (7.3%) were deleterious mutations detected *via* deleteriousness analysis, and 267 (26.7%) HR gene mutation carriers, of which 137 (13.7%) deleterious mutations detected *via* deleteriousness analysis. The frequency of HR genes was somewhat lower than that in our study, probably due to the small size of our cohort. In the OncoSG database, the number of *BRCA1/2* mutation carriers was 15 (5.0%) and there were only two (0.7%) *BRCA1/2* driver mutations. There were 35 (11.6%) HR gene mutation carriers (4.0%) and 12 deleterious mutation carriers in this database (Supplementary **Figure 1**). This frequency was significantly lower than that in our study, possibly due to the fact that only lung adenocarcinoma was present in this cohort.

In our current study, we detected an association between HR gene mutations and the response to platinum-based chemotherapy in NSCLC. However, this study was affected by several limitations. Firstly, the sample size was relatively small; secondly, all subjects were selected from a single center. These situations may have led to selection bias in the results, and may also have contributed to false negatives or positives. Therefore, a substantial amount of further work is required before HR gene mutations can be definitively used for response prediction of platinum-based chemotherapy in NSCLC. A randomized controlled trial (RCT) using a larger cohort, as well as experimental application in clinical practice may help confirm the predictive value of HR gene mutations and enhance our understanding of the mechanisms underlying NSCLC.

In brief, our results indicate that NGS profiling of HR gene mutations show potential as a tool for developing effective biomarkers that predict the efficacy of platinum-based chemotherapy in advanced and metastatic NSCLC.

## Data availability statement

Raw data generated from targeted sequencing can be found in the CNCB-NGDC (http://bigd.big.ac.cn/gsub/) with the accession number, HRA000690.

## Ethics statement

The studies involving human participants were reviewed and approved by Tianjin medical university general hospital ethics committee. The patients/participants provided their written informed consent to participate in this study.

## Author contributions

LZ analyzed the data and drafted the manuscript. FM performed patient selection and enrollment. GS collected the patient follow-up information. LT provided methodological advice. DZ contributed to study conception and design. All authors approved the submitted manuscript.

## Supplementary material

Supplementary Figure 1 Mutation frequency analysis of *BRCA1/2* and HR genes in NSCLC in public databases and in our study. **(A)** Mutation frequency of *BRCA1/2* in NSCLC in our study, TCGA database and adenocarcinoma in East Asian Cohort (OncoSG). **(B)** Mutation frequency of HR genes in NSCLC in our study, TCGA database and adenocarcinoma in East Asian Cohort (OncoSG).
