# Supplementary figures and images for: Next-generation sequencing of homologous recombination genes could predict efficacy of platinum-based chemotherapy in non-small cell lung cancer
Source: Front Oncol. 2022 Dec 14;12:1035808. doi: 10.3389/fonc.2022.1035808 (PMC9794762; doi:10.3389/fonc.2022.1035808)

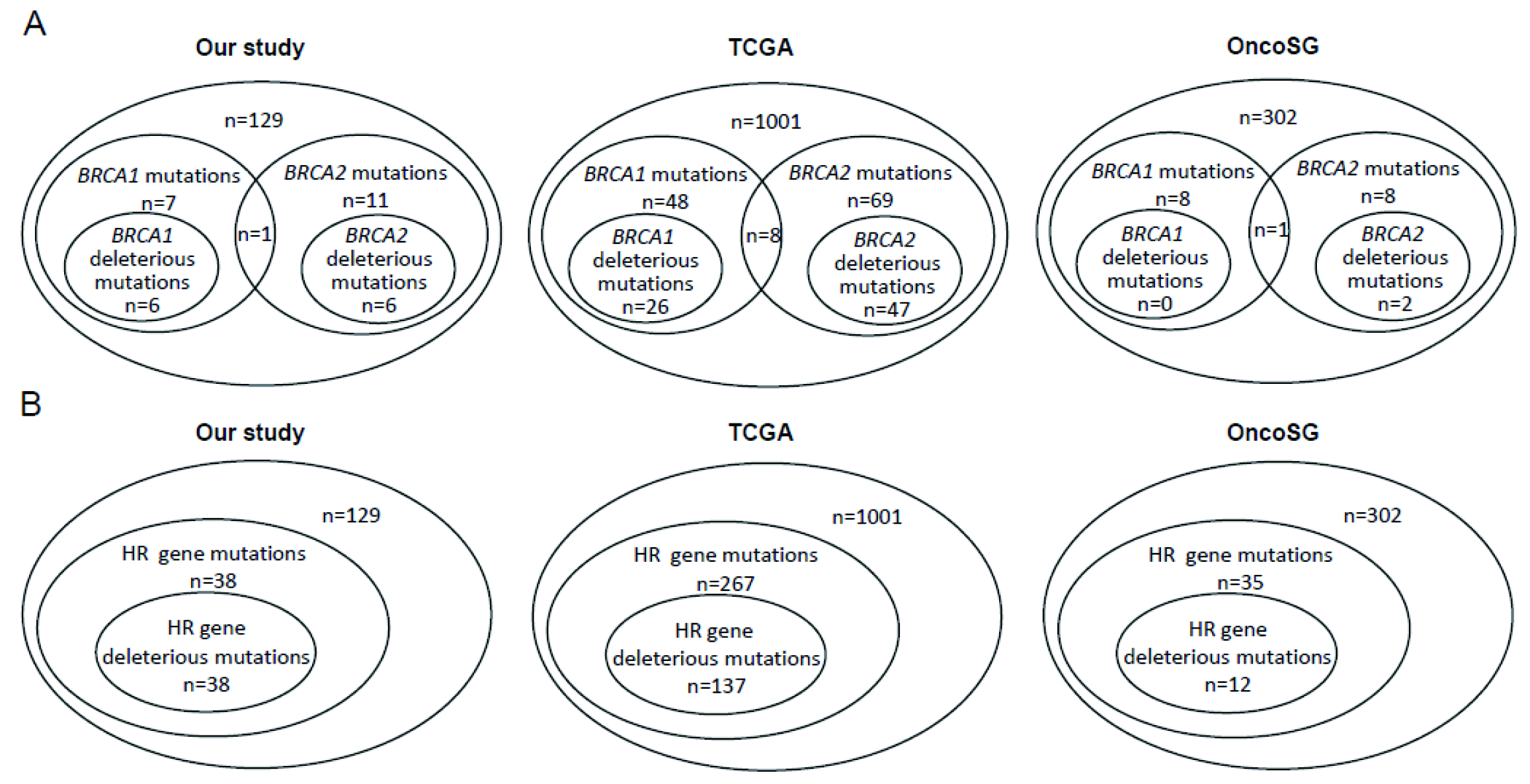

Supplement: Supplementary Figure 1 — Mutation frequency analysis of BRCA1/2 and HR genes in NSCLC in public databases and in our study. (A) Mutation frequency of BRCA1/2 in NSCLC in our study, TCGA database and adenocarcinoma in East Asian Cohort (OncoSG). (B) Mutation frequency of HR genes in NSCLC in our study, TCGA database and adenocarcinoma in East Asian Cohort (OncoSG). [file Image_1.tif]
